# Purinergic Receptor Expression and Potential Association with Human Embryonic Stem Cell-Derived Oligodendrocyte Progenitor Cell Development 

**DOI:** 10.22074/cellj.2017.3906

**Published:** 2017-08-19

**Authors:** Shirin Kashfi, Maryam Peymani, Kamran Ghaedi, Hossein Baharvand, Mohammad Hossein Nasr Esfahani, Mohammad Javan

**Affiliations:** 1 Department of Developmental Biology, University of Science and Culture, Tehran, Iran; 2 Department of Cellular Biotechnology, Cell Science Research Center, Royan Institute for Biotechnology, ACECR, Isfahan, Iran; 3 Department of Biology, Faculty of Sciences, University of Isfahan, Isfahan, Iran; 4 Department of Stem Cell and Developmental Biology, Cell Science Research Center, Royan Institute for Stem Cell Biology and Technology, ACECR, Tehran, Iran; 5 Department of Physiology, Faculty of Medical Sciences, Tarbiat Modares University, Tehran, Iran

**Keywords:** Human Embryonic Stem Cell, Oligodendrocyte Progenitor Cell, Purinergic Receptors, A_1_ Adenosine Receptor

## Abstract

**Objective:**

Due to recent progress in production of human embryonic stem cell-derived oligodendrocyte progenitor cells (hESC-OPCs) for ameliorating myelin disease such as multiple sclerosis (MS) and the role of purinergic signaling in OPCs development, we avaluated the profile of purinergic receptors expression during development of OPCs from hESC.

**Materials and Methods:**

In this experimental study, we used reverse transcription and quantitative polymerase chain reaction (RT-qPCR) to obtain more information about potential roles of purinergic receptors during in vitro production of hESC-OPCs. We first determined the expression level of different subtypes of purinergic receptors in hESCs, embryoid bodies (EBs), and hESC-OPCs. The effects of A1adenosine receptor (A_1_AR) activation on hESC-OPCs development were subsequently examined.

**Results:**

hESCs and OPCs had different mRNA expression levels of the AR subtypes. ARs mRNA were expressed in the EB stage, except for A_2A_AR. We observed expressions of several *P2X (P2X_1, 2, 3, 4, 5, 7_)* and *P2Y (P2Y_1, 2, 4, 6, 11-14_)* genes in hESCs. hESC-OPCs expressed different subtypes of P2X (P2X_1, 2, 3,4,5,7_) and P2Y (P2Y_1, 2, 4, 6, 11-14_). Except for *P2X_1_* and *P2X_6_*, all other P2X and P2Y purinergic receptor subtypes expressed in EBs. We also indicate that A_1_AR might be involved in modulating gene expression levels of cell cycle regulators in an agonist and/or dose-dependent manner.

**Conclusion:**

Elucidation of the expression pattern of purinergic receptors and the effects of different subtypes of these receptors in hESC-OPCs may have a promising role in future cell-based therapy or drug design for demyelinating disease.

## Introduction

Oligodendrocytes are responsible for synthesis and maintenance of myelin sheaths around the axons as well as providing trophic support for axons in the central nervous system (CNS) (1, 2). Consequently, an aberration in the function of oligodendrocytes which occurs under different pathological conditions causes detrimental neurological disorders (3-5). The oligodendrocyte progenitor cells (OPCs) in adult brains are believed to serve as potential sources for the generation of mature oligodendrocytes which replace lost oligodendrocytes and remyelinate bared axons (6). However, due to local obstacles against endogenous OPCs proliferation, migration or differentiation, adult remyelination is insufficient in demyelinating neurological disorders such as multiple sclerosis (MS) (7, 8). Therefore, efforts have been directed to replace lost cells and enhance endogenous remyelination by transplanting OPCs from different sources (9, 10) or by potentiating endogenous OPCs for remyelination and functional recovery (11).

Several studies have stated that axonal release of purines (ATP or adenosine) occurs during neuronal activity (12, 13), which may promote myelination in the CNS (12, 14). It has been also reported that stimulation of purinergic signaling enhanced remyelination in a rodent model of MS; however, the exact mechanism was unknown (15). Membrane receptors, primarily classified as P1 and P2, mediate the biological effects of purine nucleotides and nucleosides. P1 G-protein coupled receptors are selective for adenosine and are usually called adenosine receptors (AR). They include four subtypes: A1, A2A, A2B and A3 (16). ATP and its derivatives mainly act through the P2 receptors which exist as two distinct families: the *P2X* ligand-gated ionotropic receptors and the *P2Y* G-protein coupled receptors. There are seven subtypes of *P2X* receptors *(P2X_1-7_)* and eight subtypes of *P2Y* receptors (*P2Y_1, 2, 4, 6, 11-14_*) (17). However, only few studies have addressed the expression of particular purinergic receptor subtypes in isolated rodent OPCs and discuss probable roles for purines on oligodendrocyte development (12, 18, 19).

The increasing need for pluripotent stem cell derived-OPC replacement therapy and multiple roles of purines and purinergic receptors in the CNS have prompted us to perform a comprehensive study of the pattern changes of purinergic receptor mRNA expression during differentiation of hESC to OPCs. In this regard, we can characterize mRNA expression profiles of these receptors in the human embryonic stem cell (hESC) line RH6 and cell aggregates, known as embryoid bodies (EBs) which resemble an early stage of normal development (20). This data can provide a valuable resource for future studies on the effects of the purinergic system during hESC differentiation to oligodendrocyte lineage cells. According to researchers, a focus on characterization of the physiological state of ESC derived cells can improve the success of cell-based therapies (21). We have attempted to demonstrate the effects of A_1_AR activation in hESC-derived OPC (hESC-OPCs) developmental processes such as proliferation and differentiation *in vitro*. To the best of our knowledge, this is the first report that presents a profile of purinergic receptor expression in hESCs and derivatives during OPC production and the role of A_1_AR signaling in hESC-OPCs.

## Materials and Methods

Chemicals were purchased from Gibco (USA) or Sigma-Aldrich (USA) unless indicated otherwise. Materials purchased from Sigma- Aldrich included platelet derived growth factor-AA (PDGF-AA), epidermal growth factor (EGF), triiodothyronine (T3), Matrigel, all-trans retinoic acid (RA), paraformaldehyde, Triton, 4’,6-diamidino-2-phenylindole (DAPI), 5-bromo-2´-deoxyuridine (BrdU), and N6- cyclopentyladenosine (CPA). Materials purchased from Gibco included DMEM/ F12, insulin-transferrin-selenium (ITS), N2 supplement, fetal bovine serum (FBS), penicillin/streptomycin, L-glutamine, nonessential amino acids (NEAA), and B27 supplement. We purchased 5´-Chloro-5´- deoxy-endo-2-norbornyl adenosine (5´Cl5´d- (±)-ENBA) from Tocris and 8-Cyclopentyl-1 3-dipropyl xanthine (DPCPX) from Abcam.

### Derivation of oligodendrocyte progenitor cells from human embryonic stem cells

**Fig.1:**
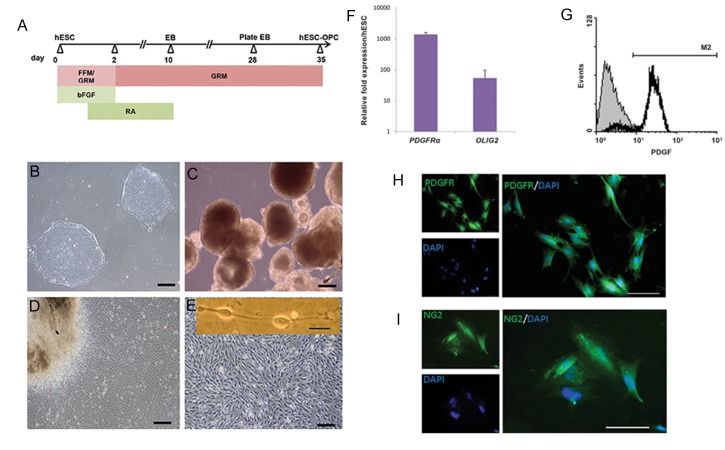
Different stages of human embryonic stem cell (hESC) differentiation into oligodendrocyte progenitor cells (OPCs) and characterization of hESC-OPCs. A. Schematic presentation of the steps for hESC differentiation into OPCs as described in the materials and methods section, B. Undifferentiated hESC colonies, C. hESC-derived embryoid bodies (EB), D. Plated EB, E. hESC-OPCs (scale bars: 200 μm, insert in E: 50 μm), F. mRNA expression levels of platelet-derived growth factor-α (*PDGFRα*) and oligodendrocyte lineage transcription factor 2 (*OLIG2*) in cultured hESc-OPCs. Data is expressed as mean + SEM, G. hESC-OPCs recognized by the cell surface marker PDGFRα according to flow cytometry analysis. Immunostaining for H. PDGFRα, and I. NG2 surface markers expression in hESCOPCs. Green; PDGFRα or NG2, blue; DAPI (scale bar: 50 μm). bFGF; Basic ﬁbroblast growth factor, FFM; Feeder-free media, GRM; Glial restriction media, and RA; Retinoic acid.

For this experimental study, the RH6 hESC line was obtained from Royan Institute (Iran). hESCs were maintained and expanded under feeder-free culture conditions in the presence of 300 ng/ml of human basic ﬁbroblast growth factor (bFGF, Royan Institute, Iran) using a previously described protocol (22). hESCs were differentiated according to a published protocol (23) with some modifications. Briefly, dissociated colonies were placed in low attachment dishes in 50% feeder-free media (FFM) and 50% glial restriction media (GRM) that contained 20 ng/ml EGF for 2 days. On day 1 the media contained 300 ng/ml bFGF. On day 2 it was supplemented with bFGF and 10 μM RA. This media was subsequently replaced with 100% GRM and supplemented with RA for an additional 8 days. Cells were then exposed for 18 days to GRM without RA. At day 28, yellow spheres were plated in 12- well plates coated with Matrigel for a period of 7 days. Cultures were then passaged using Accutase (Millipore, USA) and we excluded any remnant spheres. Cells were replated on Matrigel and cultured in GRM. The total time for the differentiation protocol was 35 days. Figure 1A represents a summary of the protocol used in this study.

### Cell collection and total RNA isolation

Cell collection was performed at three stages of the oligodendrocyte differentiation procedure: hESC (day 0), 10 day-old EBs, and the hESCOPC stage. All samples were collected and the total RNA extracted according to the RNeasy Mini Kit (Qiagen, Germany) procedure. RNA concentration was measured on a Biochrom WPA (Biowave, UK) spectrophotometer. The 260/280 ratio was not less than 1.8 for RNA samples included in this study.

### Reverse transcription and quantitative polymerase chain reaction

DNA was degraded with the use of a DNaseI, RNase-free kit (Takara, Japan) and cDNA was subsequently prepared with the Takara cDNA Synthesis Kit based on the manufacturer’s instructions to a final concentration of 25 ng/μl.

We determined the expression levels of all purinergic receptors, oligodendrocyte lineage transcription factor 2 (*OLIG2*), platelet-derived growth factor-α (*PDGFRα*), proteolipid protein 1 (*PLP1*), galactosylceramidase (*GALC*), cell cycle regulator including cyclin-dependent kinase inhibitor 1A (*CDKN1A*) that encodes for the p21^Cip1^ protein, cyclin-dependent kinase inhibitor 1B (*CDKN1B*) which encodes for the p27^Kip1^ protein, cyclin D1 (*CCND1*), and a housekeeping gene, glyceraldehyde 3-phosphate dehydrogenase (*GAPDH*). The PCR mixture contained 10 μl SYBR Green PCR Master Mix (Takara, Japan), 3 pmole of each primer, and 25 ng of cDNA for each reaction in a final volume of 20 μl. Specific primer pairs (Table 1) were designed by the Beacon Designer (version 7.2) and Oligo 7 primer analysis software (version 7). Detection and quantification of each sample was performed by the Applied Biosystems StepOnePlus Real- Time PCR system (ABI, USA). In order to further verify the specificity of the RT-qPCR assays, we performed each experiment with samples that lacked the cDNA template along with samples that contained positive control cDNA obtained from appropriate human tissues proven to have high expression levels of the desired genes (17, 24).

Expression levels of genes were estimated by the delta-delta Ct method. All Ct values calculated from the target genes were normalized to *GAPDH* in each sample and calibrated using calculations from each selected gene of the control sample. For expression levels of purinergic genes all normalized values were calibrated by using calculations from each selected gene of the P1 or P2 subfamily in hESCs. Each experiment consisted of at least three independent replicates for each stage and each replicate included three identical samples. The normalized calibrated value was given by the equation 2^-ΔΔCt^. Amplification products were resolved on 2% agarose gel (Invitrogen, USA), stained with ethidium bromide (Sinaclone, Iran), and the fragment sizes were determined by comparisons to known DNA standards.

**Table 1:** Reverse transcription and quantitative polymerase chain reaction (RT-qPCR) primers


Gene	Primer sequence (5ˊ-3ˊ)	Accession number	Amplicon size (bp)

*A1*	F: CTTCTTTGTGTGGGTGCT	NM_000674.2	79
	R: CTGCTTGCGGATTAGGTAG		
*A2A*	F: CCCAGAGGTGACATTTGAC	NM_000675.4	87
	R: GCAGCCAGAGAGTGAAAG		
*A2B*	F: TCAGTAGTAGGCTCCAAG	NM_000676.2	133
	R: ACCATAAACAAGGCAGAC		
*A3*	F: AAAGGCTGGGTATCGGCTGT	NM_000677.3	134
	R: AAGGAGGCAAACGGGAGAAG		
*P2X1*	F: ATCTGTGCTCTCCGATGT	NM-00558.2	98
	R: AGTTCAGCCGAGGAATTG		
*P2X2*	F: TGGGACTGTGACCTGGACCT	NM_012226.3	106
	R: ACCTGAAGTTGTAGCCTGACGAG		
*P2X3*	F: CATCCTGCTCAACTTCCT	NM-002559.3	78
	R: TTCAGCGTAGTCTCATTCA		
*P2X4*	F: CCTTCCCAACATCACCACTAC	NM_001256796.1	107
	R: GTCCTGCGTTCTCCACTATT		
*P2X5*	F: TGAATTGCCTCTGCTTACGTT	NM_001204519.1	197
	R: TCCGTCCTGATGACCCCA		
*P2X6*	F: CTTCTCTGGTGCTGTGAT	NM_001159554.1	82
	R: GGGATAGGGAGGTGGATTA		
*P2X7*	F: GCCACAACTACACCACGAGA	NM_002562.5	161
	R: GCCCATTATTCCGCCCTGA		
*P2Y1*	F: GAATCTCCAAACACCTCTCTG	NM_002563.3	175
	R: GAAAGCAAACCCAAACAAGC		
*P2Y2*	F: CTGGTAGCGAGAACACTAAGG	NM_002564.2	98
	R: GCACAAGTCCTGGTCCTCTA		
*P2Y4*	F: GTGGAGCTGGACTGTTGGTT	NM_002565.3	106
	R: ATAGGGTTGGGGCGTTAAGG		
*P2Y6*	F: AAACCATGCGGAGAATTAGAG	NM_004154.3	100
	R: AGAAGGGGCTGAAGAAATAGTT		
*P2Y11*	F:GACTGGAGACGCAAGAACA	NM-002566.4	100
	R: CCTTGGCGACAGAAGACA		
*P2Y12*	F: GTAAGAACGAGGGGTGTAGG	NM_022788.3	132
	R: GGTTTGGCTCAGGGTGTAAG		
*P2Y13*	F: GCCGACTTGATAATGACACT	NM- 176894.2	150
	R: TATGAGCCCTAACAGCACGAT		
*P2Y14*	F: TAGCCGCAACATATTCAGCATCG	NM_001081455.1	165
	R: GCAGCAGATAGTAGCAGAGTGA		
*PDGFRα*	F: TACACTTGCTATTACAACCACA	NM_006206.4	135
	R: ATCCTCCACGATGACTAAAT		
*OLIG2*	F: CGACTCATCTTTCCTTCTCTAA	NM_005806.3	175
	R: CGCACTTACCTCATCATTG		
*PLP1*	F: AGCATAAGGGAGCGTAGAATC	NM_176894.2	109
	R: CAAGGAGAAGGGAGTGAGAAG		
*GALC*	F: TCGTTTCCTCAGCCTCATCTC	NM_001201402.1	113
	R: CTCCCCTCCTTCCACACATAAG		
*CDNK1A*	F: AGCGACCTTCCTCATCCAC	NM_000389.4	99
	R: GCCTCTACTGCCACCATCTT		
*CDNK1B*	F: GCAACCGACGATTCTTCTACTC	NM_004064.4	109
	R: CAGGCTTCTTGGGCGTCT		
*CCND1*	F: GCGGAGGAGAACAAACAG	NM_053056	179
	R: TGTGAGGCGGTAGTAGGA		
*GAPDH*	F: CCACTCCTCCACCTTTGACG	NM_002046.3	107
	R: CCACCACCCTGTTGCTGTAG		


### Proliferation and apoptosis assays

We used the BrdU incorporation assay to evaluate the fraction of hESC-OPC that underwent proliferation *in vitro*. hESC-OPCs were first cultured in 12-well plates (3×10^4^ cells/cm^2^) and synchronized. Then, cells were exposed to GRM medium that contained A_1_AR selective agonists (0.5 μM CPA or 1 μM 5´Cl5´d- (±)-ENBA) for 48 hours. For the BrdU assay, hESCOPCs were labeled with BrdU at concentration of 10 μM overnight before the study was terminated. Cells were fixed and immunocytofluorescence staining performed according to manufacturer protocols and counterstained with DAPI (3 ng/ml) for 5 minutes. Proliferation rate was calculated as the ratio of BrdU/ DAPI+ nuclei per microscopic fields. A total of 1000 cells per coverslips were sampled to obtain a mean for each well.

Plasma membrane binding of annexin V (IQ product) was used to detect and quantify apoptotic hESC-OPCs after they were treated with A_1_AR selective agonists for 48 hours with respect to untreated cells according to the manufacturer’s protocol. Detached cells were collected by centrifugation and resuspended in annexin V binding buffer. Then, cells were incubated on ice with 10 μl of annexin V-FITC for 20 minutes. In order to discriminate between apoptotic and dead cells, propidium iodide (PI) was used for 10 minutes at room temperature. Cells were analyzed and quantitated by flow cytometry.

### Differentiation assay

For differentiation studies, we incubated the cells with GRM that contained growth factors and 0.5 μM CPA or 1 μM 5´Cl5´d-(±)-ENBA for 48 hours. RT-qPCR expression levels of the cell cycle regulator genes *CDKN1A* and *CDKN1B* which encode p21^Cip1^ and p27^Kip1^, two cell cycle dependent kinase inhibitors (CDKIs), and *CCND1* which encodes cyclin D1 (a regulator of G1 cyclin dependent kinases) have been compared with untreated cells. mRNA expression levels of *PLP1* and *GALC*, two markers of oligodendrocyte lineage cells, were evaluated to determine the differentiation stages of treated and untreated cells.

### Immunocytofluorescence

For immunocytofluorescence, cells were washed twice with PBS and fixed with 4% paraformaldehyde in PBS for 10 minutes. Permeabilization was carried out either with 0.1% Triton X-100 for 15 minutes for PDGFRα and BrdU or 0.05% Triton X-100 for 30 minutes for NG2. Cells were washed twice and incubated overnight at 4˚C with primary antibody. The primary antibodies included rabbit anti-PDGFRα (1:200, Cell Signaling, USA), rabbit anti-NG2 (1:200, Millipore, USA), and mouse anti-BrdU (1:750, Sigma, USA). Cells were washed three times, then incubated with secondary antibodies in 5 mg/ ml bovine serum albumin (BSA) at 37˚C for 1 hour and rinsed three times. The secondary antibody was goat anti-rabbit FITC (1:80, Sigma, USA) and Alexa fluor 568 goat anti-mouse IgG (1:300, Invitrogen, USA). The negative controls consisted of matched isotype controls. The nuclei were stained with DAPI. The stained cells were analyzed with a fluorescent microscope (Olympus, Japan) and images acquired with an Olympus DP70 camera (Olympus, Japan).

### Flow cytometry

Analysis of hESC-OPCs was performed by a FACS Calibur flow cytometer (Becton Dickinson, USA) with a 488 nm argon laser. Briefly, the cells were dissociated with accutase (Millipore, USA) at 37˚C for 5 minutes. Then, cells were washed twice with PBS by centrifugation at 1500 rpm for 10 minutes. The cells were fixed with 4% paraformaldehyde in PBS for 10 minutes. After washing twice with PBS, the cells were permeabilized with 0.1% triton X-100 for 15 minutes. Then, cells were washed twice and triturated with a narrow glass Pasteur pipette to prepare a single cell suspension. The primary antibody, rabbit anti-PDGFRα (1:200, Cell Signaling, USA), was added to the cells and the suspension was allowed to incubate at 37˚C for 2 hours. A secondary antibody, goat anti-rabbit IgG-FITC (1:50, Chemicon, USA), was added to the cells, after which they were incubated at 37˚C for 45 minutes. The negative control was the sample without primary antibodies. Analysis of annexin V/PI staining by flow cytometry was performed as previously described. A forward and side scatter gate was used to select target cells from the aggregates. We calculated a total of 10000 events for each sample with data analysis by WinMDI 2.9 software. Green fluorescence was detected by the FL1-H detector and displayed in the histogram.

### Statistical analysis

Statistical analysis was performed using either ANOVA followed by a multiple comparison *post hoc* Tukey’s test for purinergic receptor expression analysis or the student’s t test for other analyses. SPSS (version 17) was used to express data as means ± SEM obtained from three independent experiments. A value of P<0.05 was considered statistically significant.

## Results

### Differentiation and characterization of human embryonic stem cells to oligodendrocyte progenitor cells

Previous work has shown that hESCs can be efficiently differentiated into OPCs through defined stages (23). We began differentiation of OPCs by culturing hESCs in a suspension to induce EB formation. For further differentiation, we chose EBs that had adequate morphologies and seeded them (Fig .1A). After 25 days, most cells exhibited a typical OPC morphology characterized by small bipolar cells (25). The morphology of cells in different stages is illustrated in Figure 1B-E. RT-qPCR analysis indicated that hESC-OPCs expressed high levels of *PDGFRα* and *OLIG2* genes (Fig .1F). In order to further confirm the success of OPC differentiation, we examined the expression of PDGFRα, a surface marker for OPCs, at the protein level by flow cytometry (Fig .1G) and immunostaining (Fig .1H). Flow cytometry analysis indicated that approximately 90% of our cells were PDGFRα positive. These cells also expressed nerve-glial antigen 2 (NG2) sulfated proteoglican, another OPC surface marker, as confirmed by immunostaining (Fig .1I).

### P1 receptor subfamily mRNA expression in human embryonic stem cells, embryoid bodies, and human embryonic stem cell-derived oligodendrocyte progenitor cells

**Fig.2:**
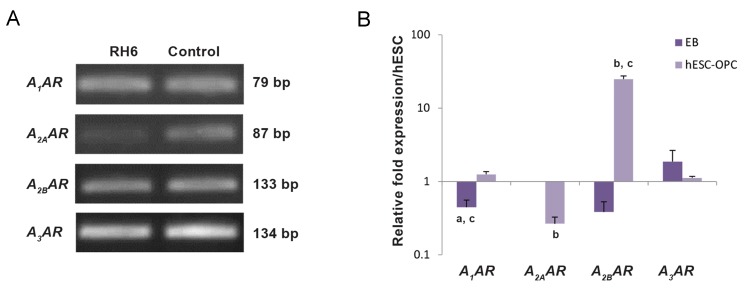
Different levels of the P1 receptor subfamily mRNA expressions in human embryonic stem cells (hESCs), embryoid bodies (EBs), and hESC-derived oligodendrocyte progenitor cells (hESC-OPCs). A. Reverse transcription and quantitative polymerase chain reaction (RT-qPCR) products obtained from hESCs and separated on gel agarose and B. The profile of P1 receptor mRNA expression in EBs and hESC-OPCs as examined by RT-qPCR. RT-qPCR was performed as described in the materials and methods section. Bars represent the mean of triplicate independent experiments ± SEM. a, b, and c indicate significant differences between hESCs and EBs, hESCs and hESCOPCs, hESC-OPCs and EB samples, respectively at P<0.05.

We used RT-qPCR to determine the level of mRNA expression in four different subtypes of P1 receptors. Gene expression analysis revealed that all subtypes of the P1 receptor family *A_1_AR, A_2A_AR, A_2B_AR,* and *A_3_AR* were present in hESCs, albeit with different degrees of expression (Fig .2A). The level of *A_1_AR* and *A_2B_AR* mRNA decreased significantly in the EB stage compared to hESCs (P<0.05) but *A_3_AR* showed the highest level of expression in EBs. Cells in this stage were negative for *A_2A_AR*. In hESC-OPCs, the mRNA of target genes *A_1_AR, A_2A_AR, A_2B_AR* and *A_3_AR* could be detected, although the expression level of *A_2A_AR* decreased and *A_2B_AR* mRNA expression increased significantly in hESC-OPCs compared to hESCs or EBs (P<0.05, Fig .2B).

### *P2X* receptor subfamily mRNA expression in human embryonic stem cells, embryoid bodies, and human embryonic stem cell-derived oligodendrocyte progenitor cells

**Fig.3:**
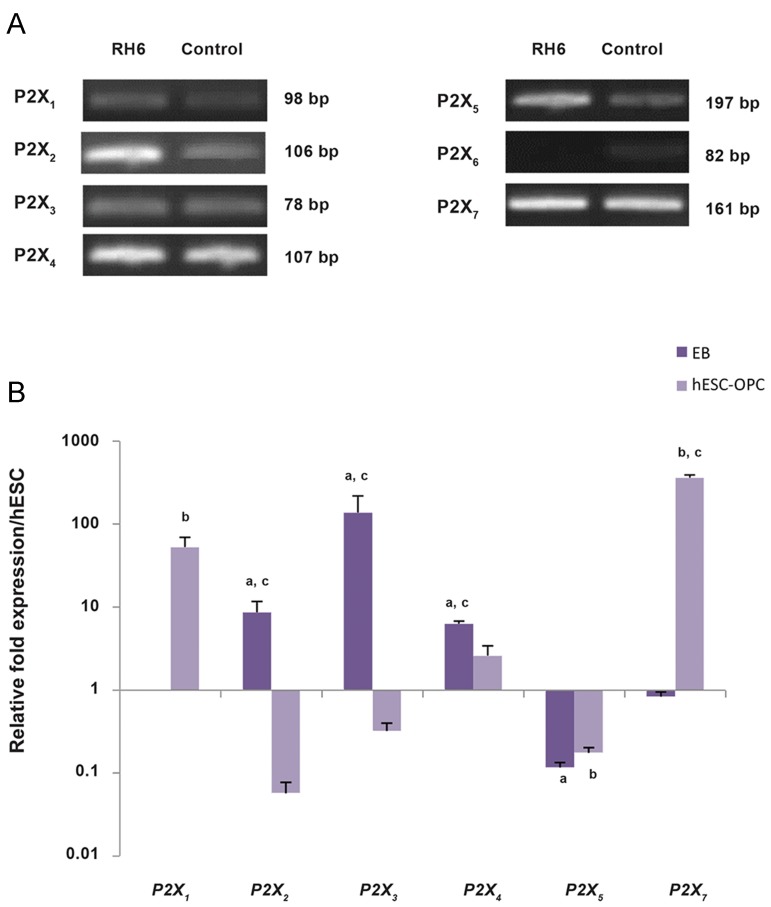
Different levels of *P2X* receptor subfamily mRNA expression in human embryonic stem cells (hESCs), embryoid bodies (EBs), and hESC-derived oligodendrocyte progenitor cells (hESC-OPCs). A. Reverse transcription and quantitative polymerase chain reaction (RTqPCR) products obtained from hESCs and separated on gel agarose, B. The profile of *P2X* receptor mRNA expression in EBs and hESCOPCs as examined by RT-qPCR. RT-qPCR was performed as described in the materials and methods section. Bars represent the mean of triplicate independent experiments ± SEM. a, b, and c indicate significant differences between hESCs and EBs, hESCs and hESC-OPCs, and hESC-OPCs and EB samples respectively at P<0.05.

Figure 3A shows the mRNA expression levels of the *P2X* subfamily receptors in hESCs. *P2X_1_* did not express in EBs, but significantly up-regulated in hESC-OPCs compared to undifferentiated hESCs (P<0.05). We observed a significant increase in the expression level of *P2X_2_, P2X_3_,* and *P2X_4_* in EBs compared to hESCs, whereas *P2X_5_* had a significant downregulation in this stage (P<0.05). Comparative analysis of mRNA expression levels of these receptors in hESC-OPCs showed downregulation of them compared to their expression levels in hESCs. Expression of the *P2X_4_* receptor in hESC-OPCs showed a non-significant increase compared to hESCs (P>0.05). The current data showed that we had no *P2X_6_* expression in any of the cell populations. Interestingly, *P2X_7_* had the highest expression in hESC-OPCs, but its expression did not show significant changes during OPC differentiation (P>0.05). Our data confirmed the expression of all subtypes of *P2X* receptors except for *P2X_6_* in hESC-OPCs (Fig .3B).

### *P2Y* receptor subfamily mRNA expression in human embryonic stem cells, embryoid bodies, and human embryonic stem cell-derived oligodendrocyte progenitor cells

**Fig.4:**
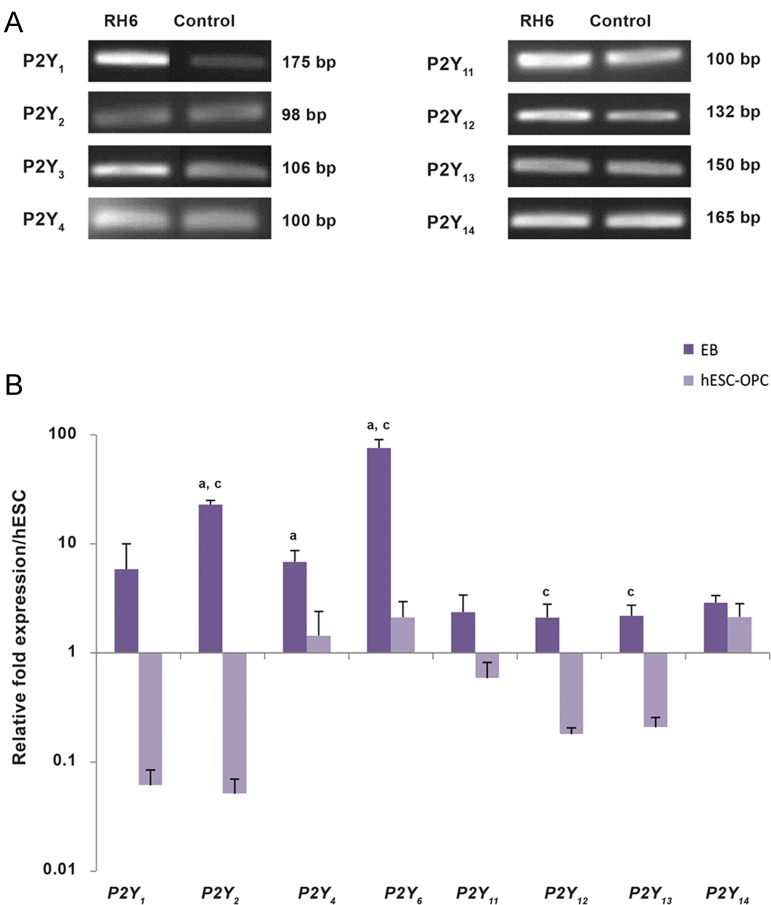
Different level of *P2Y* receptor subfamily mRNA expression in human embryonic stem cells (hESCs), embryoid bodies (EBs), and hESC-derived oligodendrocyte progenitor cells hESC-OPCs. A. Reverse transcription and quantitative polymerase chain reaction (RT-qPCR) products obtained from hESCs and separated on gel agarose, B. The profile of *P2Y* receptor mRNA expression in EBs and hESC-OPCs as examined by RT-qPCR. RT-qPCR was performed as described in the materials and methods section. Bars represent the mean of triplicate independent experiments ± SEM. a, b, and c indicate significant differences between hESCs and EBs, hESCs and hESC-OPCs, and hESCOPCs and EB samples respectively at P<0.05.

Figure 4A shows the results of *P2Y* receptor mRNA expression analyses in hESCs. hESCs expressed *P2Y_1_, P2Y_2_, P2Y_4_, P2Y_6_, P2Y_11_, P2Y_12_, P2Y_13_, and P2Y_14_.* OPCs differentiated from hESCs expressed all types of *P2Y* receptor subtypes at the transcriptional level without any significant change compared to hESCs (P>0.05). In the EB stage, all *P2Y* receptors showed a trend for increased expression, with the most significant increase observed for *P2Y_2_* and *P2Y_6_* receptors compared with hESCs (P<0.05). There were no significant changes observed between the expression levels of *P2Y_11_, P2Y_12_, P2Y_13_,* and *P2Y_14_* receptors (P>0.05, Fig .4B). Of note, the expression levels of all *P2Y* receptor subtypes down-regulated when cells differentiated to OPCs.

### Effects of A1 adenosine receptor activation on human embryonic stem cell-derived oligodendrocyte progenitor cell proliferation

**Fig.5:**
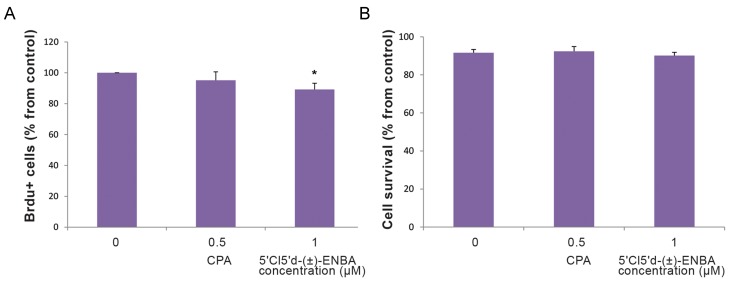
The percentage of proliferative and surviving of human embryonic stem cell-derived oligodendrocyte progenitor cells (hESCOPCs) after treatment with CPA (0.5 μM) or 5'Cl5'd-(±)-ENBA (1 μM) for 48 hours in each experiment. Data are obtained from BrdU incorporation and the annexin V affinity assay as described in the materials and methods section. A. The percentage of BrdU+ cells from the control (n=3, 40 random fields, 1000 cells per coverslip) and B. The percentage of total surviving cells. Bars represent the mean of the experiments performed in triplicate ± SEM. *; Significant differences between untreated and treated groups at P<0.05.

We examined the effect of *A_1_AR* activation on hESC-OPCs by selective *A_1_AR* agonists, CPA (0.5 μM) and 5´Cl5´d-(±)-ENBA (1 μM), for 48 hours on proliferation rate of hESC-OPCs in the presence of growth factors by using BrdU incorporation assays. As shown in Figure 5A, the percentage of BrdU+ cells did not significantly change between control and CPA treated cells (P>0.05), while the number of BrdU+ cells decreased significantly after 5´Cl5´d-(±)-ENBA treatment (P<0.05). Nonetheless, the annexin V assay showed no significant difference in cell survival between the different groups (P>0.05, Fig .5B).

### Effects of A1 adenosine receptor activation on human embryonic stem cell-derived oligodendrocyte progenitor cell differentiation

**Fig.6:**
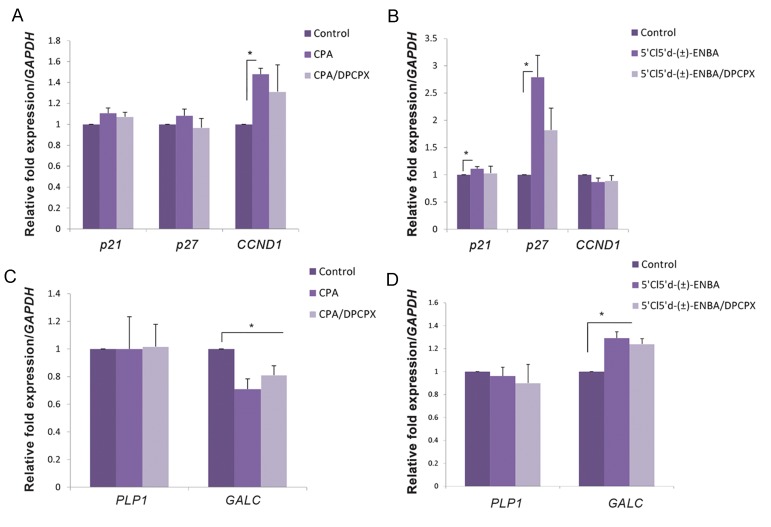
The mRNA expression level of three cell cycle regulators (*p21^Cip1^, p27^Kip1^, CCND1*), and two genes that represent oligodendrocyte lineage cell markers [proteolipid protein 1 (*PLP1*) and galactosylceramidase (*GALC*)] in human embryonic stem cell-derived oligodendrocyte progenitor cells (hESC-OPCs). Data obtained after 48 hours of treatment with CPA (0.5 μM) or 5'Cl5'd-(±)-ENBA (1 μM) and was compared with the control as measured by RT-qPCR (described in the materials and methods section). A, B. Relative fold expression level of *p21^Cip1^, p27^Kip1^, CCND1, C* and *D.* Relative fold expression level of PLPL1 and *GALC*. Bars represent the mean of independent experiments performed in triplicate ± SEM. *; Significant differences between untreated and treated groups at P<0.05.

Although, it is not sufficient, it is necessary for OPCs to exit from cell cycle when they start to differentiate (26). We examined the mRNA expression level of certain cell cycle regulators after 48 hours of treatment with CPA (0.5 μM) and 5´Cl5´d-(±)-ENBA (1 μM) compared with untreated hESC-OPCs. We chose cyclin D1, p21^Cip1^, and p27^Kip1^ because they have a critical role in regulation of OPC development (27-29). Figure 6A and B represents the results of *p21^Cip1^, p27^Kip1^,* and *CCND1* gene expressions. We have observed increased levels of *p21^Cip1^* and *p27^Kip1^* expressions after treatment with both A_1_AR selective agonists. However, 5´Cl5´d-(±)- ENBA significantly upregulated the expression of both cell cycle-dependent kinase inhibitors (P<0.05). The expression level of *CCND1* upregulated significantly after treatment with CPA (P<0.05), however we did not observe this finding for 5´Cl5´d- (±)-ENBA (P>0.05).

In order to determine to which extent changes in expression levels of the cell cycle regulators link to differentiation of hESC-OPCs, we analyzed the gene expression levels of *PLP1* and *GALC* (Fig .6C, D). *PLP1* expression level did not changed significantly after treatment with both A_1_AR agonists (P>0.05), although we observed slight downregulation of *PLP1* after 5´Cl5´d-(±)-ENBA (1 μM) treatment. Interestingly, CPA (0.5 μM) significantly decreased the level of *GALC* mRNA expression while significant increase in *GALC* expression was seen after 5´Cl5´d-(±)-ENBA (1 μM) treatment (P<0.05). The selectivity of *A_1_AR* agonist action in each experiment was determined as DPCPX (0.5 or 1 μM) antagonized the effects of CPA (0.5 μM) or 5´Cl5´d- (±)-ENBA (1 μM) respectively.

## Discussion

Oligodendrocyte dysfunction and myelin damage lead to diseases such as MS, one of the most debilitating neurological disorders (30). Experimental models of demyelinating disorders show that myelin regeneration is mainly mediated by OPCs (31, 32). Observations in MS patients have shown that OPCs present in the lesion area could not effectively differentiate and contribute to the remyelination process (33). Purinergic signaling which is believed to play a potential role in early development of organs (34, 35), including the CNS (36), may be a possible solution for enhancing differentiation capacity of endogenous or exogenous OPC.

In the current study, among the four subtypes of *AR, A_1_AR* mRNA had the highest expression in hESCs followed by A_2B_AR, A_3_AR, and A_2A_AR. Our data also revealed that the expressions of A_1_AR and A_2B_AR significantly down-regulated in the EB stage compared to undifferentiated hESCs. A_2A_AR mRNA had no expression in the EB stage, whereas we observed up-regulated expression of A_3A_AR. The increase or decrease in the expression level of these receptors possibly indicated that they might be involved in the early stages of differentiation.

Assessment of the expression pattern of P1 receptors in hESC-OPCs showed that the expressions of *A_2B_AR, A_1_AR,* and *A_3_AR* moderately up-regulated in these cells compared to hESCs or the EB stage; however, this difference was statistically significant only for A_2B_AR. Unlike *A_2B_AR,* the *A_2A_AR* expression down-regulated in hESC-OPCs compared to hESCs. Consistently, the expression of all subtypes of P1 receptor subtypes was previously reported in rodent OPCs (12). The observed enhanced expression of A_2B_AR in hESCOPCs was consistent with bioinformatics data on the expression of the A_2B_AR transcript in mice at the neurulation stage (http://www.ncbi.nlm.nih. gov/uniGene).

Our results revealed that hESCs expressed *P2X_1_, P2X_2_, P2X_3_, P2X_4_, P2X_5_,* and *P2X_7_* receptors but not the *P2X_6_* receptor. In addition, these cells expressed all *P2Y* subtype receptors at the transcriptional level. Previously, RT-PCR has been used to verify the expressions of *P2X_3_, P2X_4_, P2Y_1_,* and *P2Y_2_* receptors in mice ESCs and pharmacological assays demonstrated that ATP acted on P2 receptors which increased proliferation of mouse ESCs (37). It has been demonstrated that in human hiPSC and hESC lines, aberrational expression of the housekeeping gene hypoxanthine guanine phosphoribosyl transferase (an enzyme involved in purine biosynthesis) led to down-regulation of *P2Y_1_* expression which caused abnormal development of the dopaminergic pathway (38). These observations showed the complexity and importance of studying purinergic receptor expressions during early developmental stages.

The comparative expression profile of *P2X* receptors in EB stage and ESCs revealed that *P2X_2_, P2X_3_* and *P2X_4_* but not *P2X_7_* transcriptionally upregulated. RT-qPCR analysis showed that although all *P2Y* receptor subtypes were transcriptionally active in the EB stage, *P2Y_6_* had the highest level of expression. Changes in gene expression levels of certain types of purinergic receptors have been previously shown *in vitro* in the course of neural differentiation (39) or during early development, *in vivo* (35, 40). Of note, we studied expression of P2 receptor subtypes in the EB stage after RA treatment. Expression levels of P2 receptors have been frequently reported to be regulated by RA, a well-known morphogen agent (41, 42).

In the present study, *P2X_1_, P2X_4_,* and *P2X_7_* upregulated in hESC-OPCs whereas other genes, *P2X_2_, P2X_3_*and *P2X_5_* down-regulated. Our RTqPCR analysis showed the expression of all subtypes of *P2Y* purinergic receptors in these cells. However, their degrees of expression were mainly reduced relative to hESCs or the EB stage. Among this subtype, *P2Y_6_* showed the highest expression level in hESC-OPCs. Previously, cultured rat OPCs also expressed different P2X (P2X_1, 2, 3, 4, 7_) and P2Y (P2Y_1, 2, 4, 12, 13_) receptors (18). By functional analysis, the presence of P2X7 and several P2Y (P2Y_1, 2, 4, 6, 11, 13_) receptors were reported in OPCs (36). The results of the current study showed some similarities with previous studies.

Oligodendrocyte development is a complicated process that involves the interplay of numerous factors. It has been shown that AR and/or some *P2Y* receptors may be involved in oligodendrocyte progenitor differentiation in rodents (12, 18). Although the effects of purinergic receptors activation on human oligodendrocyte lineage cells development have not been investigated yet, we focused on the effects of A_1_AR activation on hESCOPC development by considering the following criteria: i. *A_1_AR* mRNA expression was seen in the first part of the current study, ii. It was demonstrated that A_1_AR activation played a prominent role in mediating neuroprotection and neuromodulatory effects of adenosine in CNS [reviewed in (43)], iii. A_1_AR activation ameliorated the severity of EAE and increased remyelination in an animal model of MS (15, 44), and iv. A_1_AR agonists often affect cardiovascular function such as decreased heart rate or blood pressure (45). However, it was reported that a novel series of A_1_AR agonists did not have such unintended adverse effects (46), including 5´Cl5´d-(±)-ENBA (47).

Our data demonstrated that CPA did not significantly affect hESc-OPCs proliferation (P>0.05), which supported a previous study on rodent OPCs (48). However, the proliferation rate of these cells decreased significantly after treatment with 5´Cl5´d-(±)-ENBA. Of note, changes in agonist structure have been shown to alter the ability of A_1_AR to activate different signaling pathways with diverse potency and efficacy due to different receptor conformations. So the current study results probably present another example of "functional selectivity", which has been described as "agonist-dependent receptor signaling" (49), and needs additional in depth study. However, these results may also reflect the dose-dependent effects of selective agonists.

We proposed that these results might be due to events associated with modulation of cell cycle regulators. Hence, we have focused on expression pattern of those canonical cell cycle components which play a role in G1 progression or oligodendrocyte cell cycle exit and differentiation. This hypothesis is supported by several studies on cell cycle regulation of the oligodendrocyte lineage cells (50-53). Our results have indicated that CPA treatment up-regulated gene expression levels of *p21^Cip1^* and *p27^Kip1^* non-significantly while significantly up-regulation of *CYCLIN D1*. We observed a distinct pattern in expression profile of these cell cycle regulators after 5´Cl5´d-(±)-ENBA treatment with significantly increased *p21^Cip1^* and *p27^Kip1^* levels accompanied by non-significant downregulation of *CYCLIN D1 *expression. Cyclin D1 kinase activities decreased in G1-arrested and differentiated oligodendrocytes (52).

The Kip/Cip family of cyclin dependent kinase inhibitors (including p21^Cip1^ and p27^Kip1^) has been involved in the regulation of oligodendrocyte development. Overexpression of p27^Kip1^ increased the efficiency of oligodendrocyte differentiation from induced pluripotent stem cells (29) and an increased level of proliferated OPCs has been seen in p27^Kip1^ null mutant mice (50). p21^Cip1^ is not required for cell cycle exit, but plays a role in OPC differentiation (51). However, the complex relationship between p27^Kip1^, cyclin D1, and other cell cycle proteins such as cdk4 must be considered. Some studies have suggested that Kip/Cip CDKIs are activators of cyclin D-CDK complex assembly. Then, the cyclin D-CDK complex can sequestrate the Kip/Cip family of CDKIs from cyclin E-cdk2 complexes and allow cell cycle progression (54, 55). Other studies have suggested that high expression of CDKIs can repress CDK activity (53, 56). Considering these data, it is not surprising that we have found no significant difference between CPA treated and untreated cells in our study despite elevated CYCLIN D1 gene expression. Also, there is the same mRNA expression profile for *p21^Cip1^, p27^Kip1^,* and *CYCLIN* D1 in oligodendrocytes which has been extracted from schizophrenia patients’ brains. Patients suffer from schizophrenia face to condition that mature oligodendrocytes re-enter to cell cycle and failure to differentiate (28). In addition, a significant decrease in proliferation rate of hESC-OPC after 5´Cl5´d-(±)-ENBA treatment was in accord with significant upregulation of *p21^Cip1^* and *p27^Kip1^.* This observation supported previous studies which reported that highly expressed *p27^kip1^* could suppress CDKs (56). Upregulation of *p21^Cip1^* and, especially *p27^Kip1^*, have appeared to be part of intrinsic mechanisms which cause cell cycle arrest and possibly initiation of differentiation.

Next, we sought to determine the extent to which cell cycle gene expression changes in this system accompanied with progress in oligodendrocyte differentiation by determining the mRNA expression level of some special markers of oligodendrocyte developmental stages. PLP expression has been shown to occur very early in OPCs in the spinal cord where it plays a role in normal OPCs migration. PLP expression downregulated as cells progressed through their subsequent developmental stages and then upregulated as OPCs matured into myelinating oligodendrocytes (57). It has been reported that the *GALC* gene upregulates during oligodendrocyte differentiation (58). Analysis of the gene expression level of these mentioned markers indicated that CPA significantly decreased *GALC* gene expression while *PLP1* expression showed no significant change in respect to untreated cells. This observation could be interpreted that CPA maintained cells in the progenitor state which agreed with data obtained from cell cycle regulator analysis. OPCs have been characterized as highly motile cells. Previously, it was shown that CPA had no effect on the proliferation or differentiation rates of rodent OPCs but enhanced their migration (48). We also observed an increased level of migration in hESC-OPCs exposed to CPA (data not shown).

5´Cl5´d-(±)-ENBA treatment significantly upregulated the expression level of *GALC* as well as nonsignificant downregulation of *PLP1*. These expression patterns of oligodendrocyte lineage markers might indicate that increased P27Kip1 expression after 5´Cl5´d-(±)-ENBA treatment possibly involve triggering hESC-OPCs development progression in the next stages. It was suggested that the level of p27^Kip1^ accumulation in proliferating OPC was related to oligodendrocyte differentiation (59, 60). The current study results provided additional evidence for this assumption from hESC-OPCs. However, although 5´Cl5´d- (±)-ENBA induced upregulation of p21^Cip1^ and p27^Kip1^ was associated with upregulation of *GALC*, it does not by itself promote OPC differentiation and the probable of cell cycle arrest must be also considered with further studies. On the other hand, it could be said that a few hours exposed to an agonist might not be sufficient, but this argument was not true regarding gene expression, particularly cell cycle regulators that respond rapidly to most biological signals or some downstream A_1_AR effectors involved in OPCs differentiation (26). We have not ruled out that these results do not reflect the exact changes in protein expressions and their functions.

## Conclusion

Our study of purinergic receptor subtype expressions in hESCs, EBs, and hESC-OPCs has expanded and complemented previous studies regarding the expression and distribution of these receptors in different cells as well as different developmental stages. hESC-OPCs provide a reliable source for use in cell-based therapies. Characterizing the physiological properties of these cells provides important information about their current state and subsequent behavior upon transplantation. This can open new horizons for the treatment of neurological disorders that arise from neuronal demyelination such as MS. Characterization of these receptors on hESCOPCs also promotes development of effective new drugs, as well as designing new strategies and culture media that influence their proliferation, differentiation, and maintenance. We also provide evidences that hESC-OPCs express A_1_AR which may contribute to cell cycle regulation and lineage progression in a dose- and/or agonist-dependent manner. However, the question remains to be answered regarding the extent to which these mRNA expression levels correlate with protein expression.
